# Real-world comparison of anti-CD20 therapies: efficacy, infections, and immune profiles in a German cohort

**DOI:** 10.3389/fimmu.2026.1738865

**Published:** 2026-05-12

**Authors:** Jakob Stögbauer, Moritz Bewarder, Linda Groß, Lorenz Thurner, Klaus Fassbender, Rebecca Urschel, Einar A. Høgestøl, Gro O. Nygaard, Hanne F. Harbo, Olaf Stüve, Marc Pawlitzki, Sven G. Meuth, Martina Sester, Sergiu Groppa, Mathias Fousse

**Affiliations:** 1 Department of Neurology, Saarland University Medical Center and Saarland University Faculty of Medicine, Homburg, Germany; 2 Department of Neurology, Saarland University, Homburg, Germany; 3 Department of Internal Medicine I, Saarland University Medical Center, Homburg, Germany; 4 Center for Hematology and Oncology Bethanien, Frankfurt, Germany; 5 Department of Transplant and Infection Immunology, Saarland University, Homburg, Germany; 6 Department of Neurology, Oslo University Hospital and Institute of Clinical Medicine, University of Oslo, Oslo, Norway; 7 Department of Neurology, University of Texas Southwestern Medical Center, Dallas, TX, United States; 8 Department of Neurology, Medical Faculty and University Hospital Düsseldorf, Heinrich Heine University Düsseldorf, Düsseldorf, Germany

**Keywords:** B-cell depletion, cellular immune status, multiple sclerosis, neuroimmunological diseases, ocrelizumab, ofatumumab, rituximab, T-cell function

## Abstract

**Background:**

The use of anti-CD20 drugs has become a widespread therapeutic approach in systemic and central nervous system (CNS) neuroinflammation. Apart from the desired B-cell depletion, relevant dynamics of the humoral and cellular immune response occur. Despite the extensive utilization of these drugs, direct comparative analyses of various B-cell-depleting agents remain scarce.

**Methods:**

A total of 262 patients with neuroimmunological diseases treated with ocrelizumab, ofatumumab, or rituximab were observed over a median period of 36 months. Relapses, infection rates, and the concentration of immunoglobulins were monitored quarterly. In addition, changes in cellular immunity (differential blood count, natural killer cells, CD19^+^, CD3^+^, CD4^+^, and CD8^+^ cells) along with polyclonal T-cell function (measured by reactivity) were analyzed using multidimensional flow cytometry.

**Results:**

Annual relapse rates in both the ocrelizumab and ofatumumab groups were low: 0.11 [95 % confidence interval (CI), 0.06 – 0.15] and 0.08 (95% CI, 0.05 – 0.16), respectively. Infections occurred significantly less frequently with ofatumumab (p < 0.001). Hypogammaglobulinemia was observed more frequently and earlier in rituximab patients (p < 0.001). Ocrelizumab treatment was associated with a reduction in the proportion of total lymphocytes and an increase in the proportion of CD3^+^ T cells, while ofatumumab was linked to a rise in the CD4/CD8 ratio. Anti-CD20 antibodies did not influence T-cell reactivity after polyclonal stimulation.

**Conclusions:**

B-cell depletion is effective in neuroimmunological diseases irrespective of which CD20 antibody was used. However, differences in infection rates and the occurrence of hypogammaglobulinemia were observed. Together with new insights into differences in the influence of CD20 antibodies on lymphocyte subpopulations, these findings may inform future individualized treatment strategies.

## Introduction

Interest in neuroimmunological diseases has increased significantly over the past decades. This is partly due to a better understanding of the pathophysiology of these diseases [e.g., detection of pathological autoantibodies (1, 2)], but also due to novel therapeutic approaches. In particular, reference should be made to the depletion of B cells using therapeutics directed against the CD20 antigen. CD20 is a glycosylated phosphoprotein that is expressed highly specifically on the membrane surface of B cells at different stages of maturation (3). B cells have key functions in the adaptive immune response and play a central role in the development of various autoimmune diseases. This involves antigen presentation [to T cells, among other cell types (4)] and the production of cytokines and autoantibodies (5). Consequently, drugs directed against B cells are of significant importance in the treatment of autoimmune diseases. For more than 20 years, anti-CD20-directed monoclonal antibodies, initially rituximab (RTX) (6), have been utilized in the treatment of various autoimmune diseases.

In neuroimmunology, the anti-CD20-directed antibodies ocrelizumab (OCR) and ofatumumab (OFA) have been authorized for the treatment of multiple sclerosis (MS) (7, 8). Furthermore, a multitude of additional neuroimmunological diseases are treated off-label with RTX. Depending on the underlying disease, it is highly effective in achieving disease remission (9). Its efficacy in the treatment of both relapsing (10, 11) and primary progressive (12) MS (RMS and PPMS) has also been demonstrated, despite the absence of formal approval. OCR and OFA have been demonstrated to be highly efficacious in RMS treatment (13–15). OCR is also approved for PPMS.

In addition to the desired effect on the immune system, B-cell depletion treatment (BCDT) causes an increased rate of infections (primarily of the urinary tract and upper respiratory tract), possibly depending on the duration of application (16–20). Additionally, it is evident that BCDT causes secondary hypogammaglobulinemia, but it is unclear to what extent it is considered a significant risk factor for susceptibility to infection (21, 22) (data on infection incidence and immunoglobulin levels from the pivotal trials and open-label extensions (OLE) of OCR and OFA are summarized in **Table 1**). There is not always a demonstrable link between low immunoglobulin levels and severe infections (23), especially regarding IgM (24). Therefore, it is evident that other mechanisms also appear to be involved. BCDT has been shown to not only influence immunoglobulin synthesis, but also other components of the immune system. Lymphopenia during OCR therapy occurs in approximately 20% of cases (25). Effects on T cells and natural killer (NK) cells are unclear (26–28). Moreover, functional anergy of the remaining T cells may also favor the occurrence of infections in B cell-depleted patients (29). Further studies are needed to evaluate the indirect influence of various BCDT agents on other lymphocyte subpopulations and subsequent infections.

**Table 1:** Data on infection incidence and immunoglobulin levels from the pivotal trials and open label extensions (OLE) of OCR and OFA.

	OCR		OFA	
	Pivotal trials (OPERA I*/II**) (36)	OLE (OPERA OLE) (15)	Pivotal trial (ASCLEPIOS I^#^/II^##^) (38)	OLE (ALITHIOS) (19)
Infection rates, n (%)	232/408 (56.9)* 251/417 (60.2)**	n.a.	229/465 (49.2)^#^ 259/481 (53.8)^##^	1149/1969 (58.4)
EAIR	n.a.	75.3	n.a.	41
Serum IgG below LLN, %	1.5^1^	5.4^2^	1.3^3^	1.6^5^
Serum IgM below LLN, %	16.5^1^	29.5^2^	14.3^4^	26.6^5^

OCR, Ocrelizumab; OFA, Ofatumumab; OLE, Open label extension; EAIR, Exposure-adjusted incidence rate; LLN, lower limit of normal.

^1^at week 96; ^2^at year 5; ^3^20% below LLN; ^4^10% below LLN; ^5^at year 4.

* OPERA I, * OPERA II.

# = ASKLEPIOS I; ## = ASKLEPIOS II.

Direct comparisons of the different substances are limited. Data regarding treatment effectiveness and incidence of infection in relation to the substances is only available on an occasional basis (30–32). A head-to-head analysis of therapy-associated effectiveness, infections, the dynamics of immunoglobulins, and the various immune cells over a treatment period of several years has not yet been carried out, especially one that includes data on OFA.

The objective of the study was to systematically compare the anti-CD20-targeting antibodies RTX, OCR, and OFA. To this end, we analyzed clinical events (relapses and infections) and laboratory findings (immunoglobulins, immune cell dynamics, and T cell anergy) in the long-term course of patients with MS (OCR/OFA) and other neuroimmunological diseases (RTX).

## Methods

### Patients and laboratory examination

This cohort study examined all patients treated with BCDT in the Department of Neurology at Saarland University Hospital from 2014 to 2024. According to the marketing authorization, OCR patients suffered from RMS or PPMS, while OFA patients were RMS patients only. RMS was defined as the presence of relapsing-remitting MS (RRMS) or active secondary progressive MS (aSPMS). RMS patients with an indication for BCDT were given the choice between OFA and OCR after receiving detailed information, as no data on the superiority of either drug was available at the time of the study. Patients receiving RTX in off label use had various diagnoses, including autoimmune encephalitis (AE) or cerebral vasculitis. The dosage of the drugs was chosen as follows:

- OCR: 300 mg intravenously on day 0, followed by 300 mg after 14 days, and then 600 mg or 920 mg subcutaneously every 6 months
- OFA: 20 mg subcutaneously on weeks 0, 1, 2, 4, and then monthly
- RTX: 1000 mg intravenously on day 0 and day 15, followed by semi-annual infusions

Patients had clinical visits on a 6-month basis for an average of 3 years, or more frequently if an event occurred (relapse or infection). Clinical symptoms were defined in relation to the occurrence of relapses and the Expanded Disability Status Scale (EDSS). Relapse-free survival, annual relapse rate (ARR), and EDSS were used to assess treatment efficacy. This analysis was restricted to patients with OCR and OFA, as not all RTX patients suffered from relapsing diseases or could be quantified via EDSS.

Infection was diagnosed if there were clinical symptoms (such as fever, coughing, or dysuria), and a simultaneous increase in the laboratory infection parameters (C-reactive protein (CRP) and/or leukocytosis). This was then used to calculate the exposure-adjusted incidence rate of infections per 100 patient-years (EAIR). Laboratory testing was performed on a quarterly basis, encompassing the measurement of immunoglobulins via turbidimetry and a complete white blood cell count. Furthermore, multidimensional flow cytometry was used to quantify CD3^+^, CD4^+^, CD8^+^, and CD19^+^ lymphocytes, CD4/CD8 ratios, natural killer cell counts, and polyclonal T-cell functionality. Cell counts were presented as relative frequencies (percentage of neutrophils, lymphocytes, eosinophils, basophils, and monocytes relative to total leucocyte count and the percentage of lymphocyte subset cell populations relative to total lymphocyte count).

Measurements of differential blood counts were performed using a Sysmex XN-L™ automated hematology analyzer. Lymphocyte subpopulations were assessed by flow cytometry using a BD FACSLyric™ clinical cell analyzer and the Multitest™ 6-color TBNK kit according to the manufacturer’s instructions (**Supplementary Figure 2**). Analysis of polyclonal T-cell activation and differentiation was performed from heparinized whole blood exactly as described before (33). In brief, 450 µl of whole blood was stimulated with 2.5 µg/ml of *Staphylococcus aureus* enterotoxin B (SEB; Sigma) or a negative control stimulus in the presence of 1 µg/ml of anti-CD28 and anti-CD49d costimulatory antibodies (BD Biosciences). After 2 hours, brefeldin A was added to achieve intracellular accumulation of the induced cytokines. After a further four hours, the cells were fixed and stained with fluorescently labelled antibodies towards CD4, CD69, and IFN-γ (33). At least 10,000 CD4 T cells per sample were analyzed by flow cytometry using a BD FACSCanto II and BD FACSDiva software version 6.1.3 or BD FACSLyric™ and BD FACSSuite software, respectively. Functionally active cells were quantified as a percentage of CD4 T cells coexpressing CD69 and IFN-γ as shown in the gating strategy in **Supplementary Figure 3**. Inter-assay and intra-assay variability of the assay has been shown to be low (34).

### Statistical analysis

Descriptive statistics were described using mean and standard deviation or median and range, respectively. Comparison of unpaired, non-parametric data between different groups was carried out using the Mann-Whitney U and Kruskal-Wallis test with Bonferroni correction for *post hoc*-analysis. Chi-Square test was used for categorical data.

Kaplan-Meier curves were created for survival analyses. Significant differences were tested using the log-rank test. For immune cell dynamics, median blood cell counts were analyzed separately for each substance in 3-monthly intervals (OCR, RTX up to month 48, and OFA up to month 24). Linear regression was then performed using medians to analyze the relationship between treatment period and cell count. R^2^ was used as the regression coefficient. Following this, the drug therapy’s effect on the cell count was analyzed by a moderator analysis.

Spearman’s correlation coefficient was used to analyze bivariate correlations. For multivariate analysis, multiple regression analysis and multiple binary logistic regression were used.

Statistical analysis was deemed to be significant if the p-value was less than 0.05, and confidence intervals were set at 95%.

### Software

Statistical analysis was conducted using SPSS Statistics (version 29.0.2.0) with PROCESS macro for moderation analysis (version 5.0). Graphics were created using RStudio (version 2025.05), while Microsoft Word (Microsoft 365) was used for word processing.

## Results

### Patient characteristics

A total of 262 patients (165 of whom were female) were included and followed up for a median observation time of 36 months. Demographic and clinical characteristics are shown in **Table 2**. In total, 166 patients were treated with OCR, 44 with OFA, and 52 with RTX. The OCR cohort comprised 139 patients with RMS and 27 with PPMS. The largest diagnostic groups of RTX patients were autoimmune encephalitis (n = 15) and vasculitis (n = 12). Baseline EDSS was significantly higher in OCR than in OFA patients (p < 0.001), even when only RMS patients were considered (p = 0.002). The RTX cohort was significantly older than the other groups (p < 0.001), and treatment discontinuation was significantly more frequent in these patients (p = 0.001). The predominant reason for discontinuation was clinical instability (15.5%), with infections being the most common cause in the OCR group (19.6%). Four patients developed malignancies during maintenance therapy with RTX.

**Table 2:** Patient characteristics.

	OCR	OFA	RTX	All	p*
n	166	44	52	262	
**Sex**, female, *n (%)*	107 (64.5)	33 (75)	25 (48.1)	165 (63)	**0.02**
Age at diagnosis, years, *median (range)*	33 (13; 72)	31.5 (17; 65)	54 (17; 74)	35 (13; 74)	**< 0.001**
Age at symptom onset, years, *median (range)*	32 (13; 65)	29.5 (17; 65)	54 (5; 74)	34 (5; 74)	**< 0.001**
Age at start of therapy, years, *median (range)*	46 (18; 75)	37 (18; 67)	54.5 (18; 74)	47 (18; 75)	**< 0.001**
Disease group, *n (%)*					
RMS	139 (83.7)	44 (100)	1 (2)	184 (70.2)	
PPMS	27 (16.3)			27 (10.3)	
AE			15 (28.8)	15 (5.7)	
Cerebral Vasculitis			12 (23.1)	12 (4.6)	
Other^1^			24 (46.1)	24 (9.2)	
Baseline EDSS *Median (range)*	3.5 (0 – 8)	2.25 (0.5 - 7)			**< 0.001**
Baseline EDSS (RMS only) *Median (range)*	3.25 (0 – 8)	2.25 (0.5 – 7)			**0.002**
Discontinuation of therapy, *n (%)*	51 (30.7)	7 (15.9)	26 (50)	84 (32.1)	**0.001**
Reason for discontinuation, *n (%)*					
Relapses/worsening	5 (12.8)	2 (28.5)	8 (30.8)		
Infection	10 (19.6)7	1 (14.3)	2 (7.7)	13 (15.5)	
Patient’s request	(17.9)	4 (57.1)	5 (19)	12 (14.3)	
Pregnancy	4 (10.3)		2 (7.7)	12 (14.3)	
Other adverse event	10 (19.6)		5 (19.2)	4 (4.8)	
Other^3^	3 (5.9)		4 (15.4)	12 (14.3)	
Not known	12 (23.5)			9 (10.7)	
				22 (26.2)	
Diagnosis of further autoimmune disease during treatment, *n (%)*	3 (1.8)	0 (0)	5 (9.6)	8 (3.1)	**0.007**
Diagnosis of malignoma^4^ during treatment, *n (%)*	3 (1.8)	1 (2.3)	4 (7.7)	8 (3.1)	0.093
Follow-up months*, median (range)*	39 (3; 78)	15 (3; 45)	42 (3; 117)	36 (3; 117)	

OCR, Ocrelizumab; OFA, Ofatumumab; RTX, Rituximab, RMS, relapsing multiple sclerosis, PPMS, primary progredient multiple sclerosis, AE, autoimmune encephalitis.

^1^other disease groups were Neuromyelitis optica spectrum disease (n = 5), autoimmune neuropathy (n = 4), MOGAD (n = 3), myositis (n = 3), myasthenia gravis (n = 2), paraneoplastic cerebellar degeneration (n = 2), Lambert-Eaton myasthenic syndrome (n = 1), antiphospholipid antibody syndrome (n = 1), systemic lupus erythematodes (n = 1), chronic progressive external ophthalmoplegia (n = 1), steroid responsive encephalopathy associated with autoimmune thyroiditis (n = 1);.

^2^other adverse events were allergy (n = 1), infusion reactions (n = 2), T cell defect (n = 2), macula edema (n = 1), severe hypogammaglobulinemia (n = 2), hyponatremia (n = 2), agranulocytosis (n = 1);.

^3^other reasons for discontinuations of therapy were death (not related to therapy, n = 3), malignoma (n = 3), change of diagnosis (n = 2), change to another BCDT (n = 1), dementia (n = 1);.

^4^types of malignomas were prostate carcinoma (n = 2), myeloma (n = 2), urothelial carcinoma (n = 1), endometrial cancer (n = 1), mamma carcinoma (n = 1), bronchial carcinoma (n = 1).

*P-values were calculated by Mann Whitney U test, Kruskal Wallis test, and chi square test, respectively.

Bold values are statistically significant (p < 0.05).

### Relapses and EDSS

**Figure 1:**
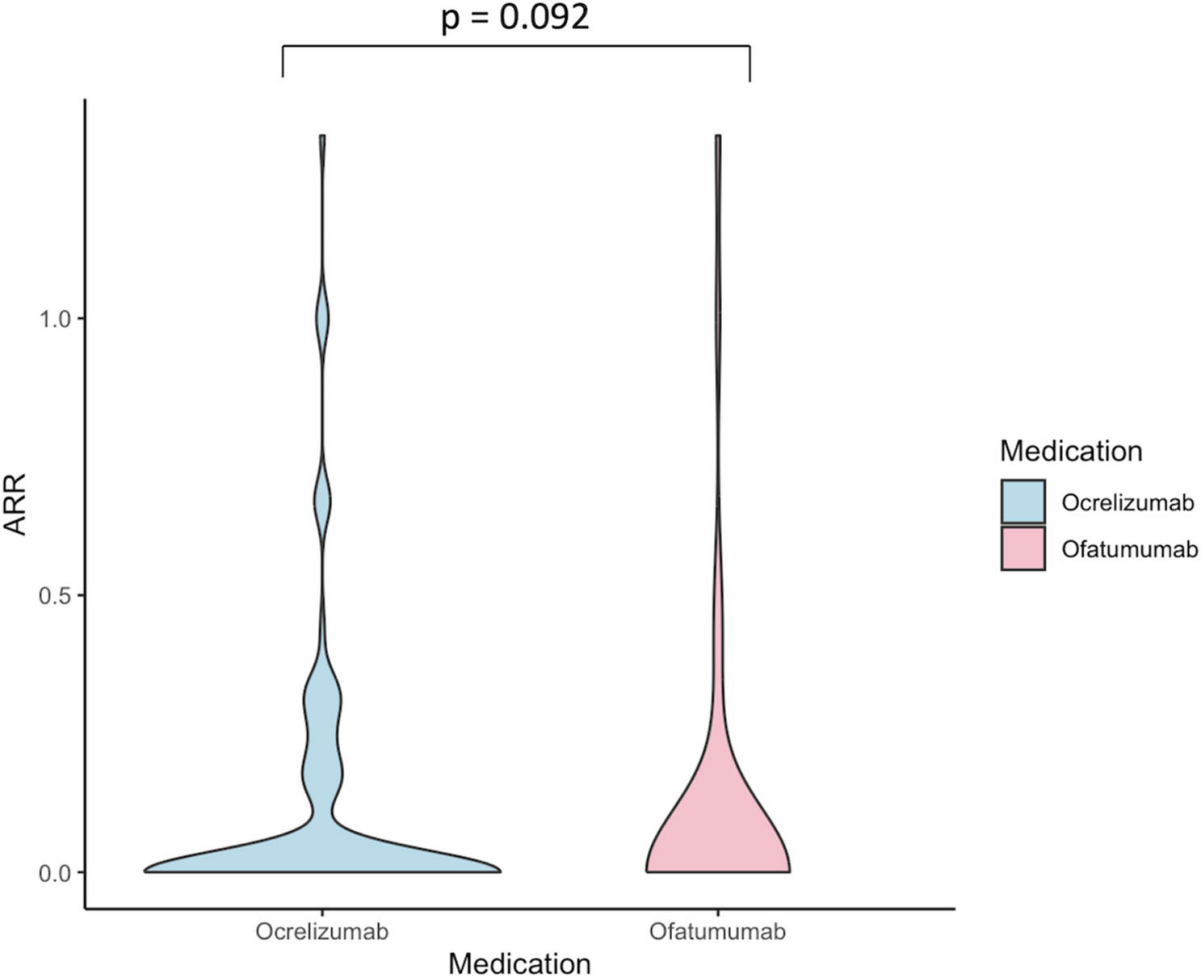
Violin plot shows annual relapse rates of Ocrelizumab and Ofatumumab. P-values calculated by use of Mann Whitney-U-test. ARR, annual relapse rate.

**Figure 2:**
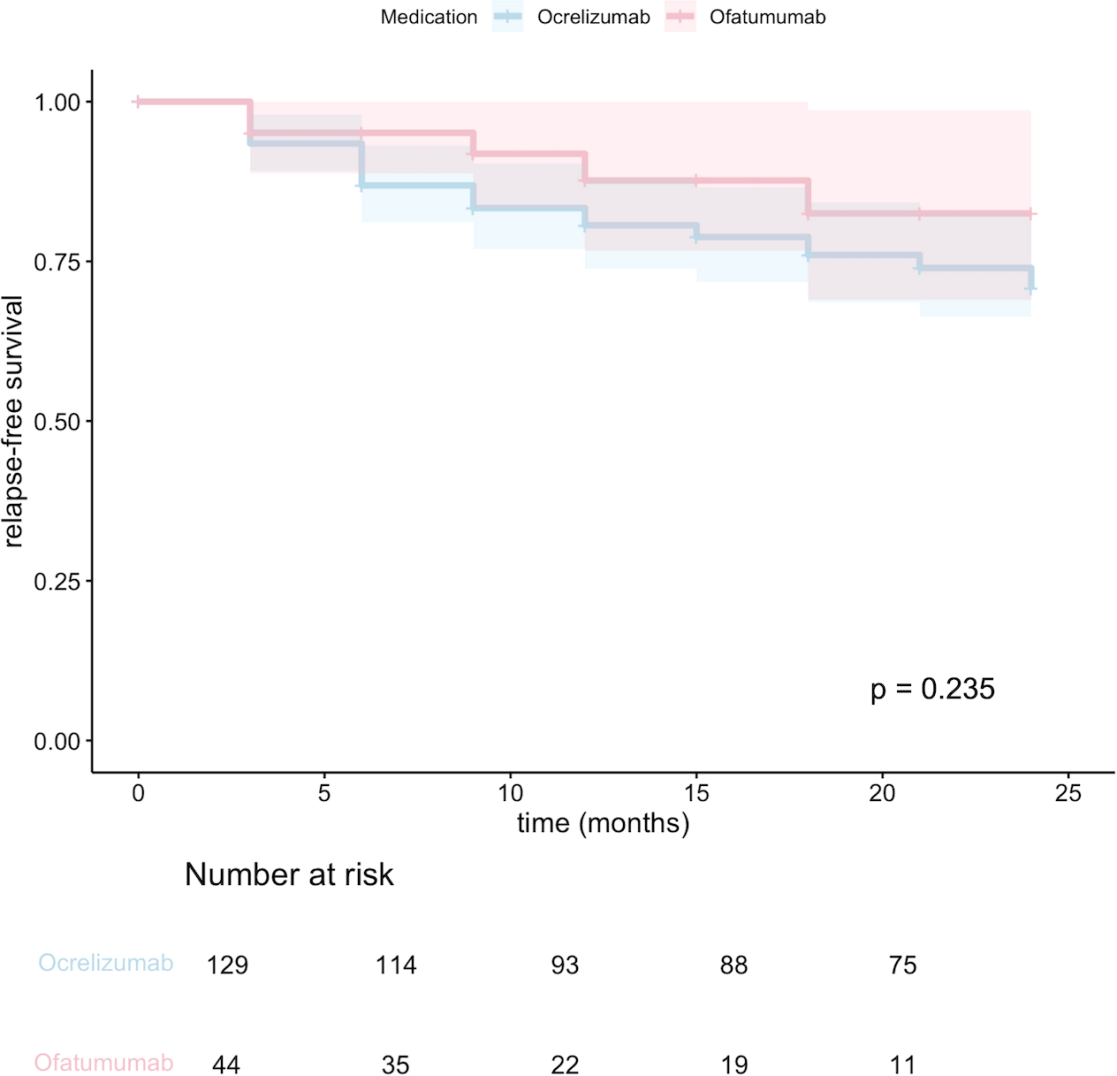
Kaplan–Meier plots indicating the proportion of RMS patients without a clinical MS relapse. Shaded areas display 95% confidence intervals. P-values were calculated by use of the log-rank test.

The occurrence of relapses and median EDSS scores were analyzed for all RMS patients in the OCR and OFA groups. PPMS patients receiving OCR were excluded from the analyses. Annual relapse rate (ARR) and relapse-free survival were used as outcome parameters. Mean ARR was found to be 0.11 (95% CI, 0.06 – 0.15) for OCR and 0.08 (95% CI, 0.05 – 0.17) for OFA with no statistically significant difference between the groups in the Mann-Whitney U test (p = 0.092, **Figure 1**). Kaplan Meier analysis also showed no significant difference in the probability of relapse-free survival between the OCR and OFA groups (p = 0.235; see **Figure 2**).

There was no association between treatment duration and relapse frequency. During the first 24 months of maintenance therapy, median EDSS in patients with OCR increased significantly from 3.5 in month 0 to 4.5 in month 24 (Spearman’s r = 0.842, p = 0.006) and from 2.25 to 3.0 in OFA patients (Spearman’s r = 0.683, p = 0.036).

### Infections

**Figure 3:**
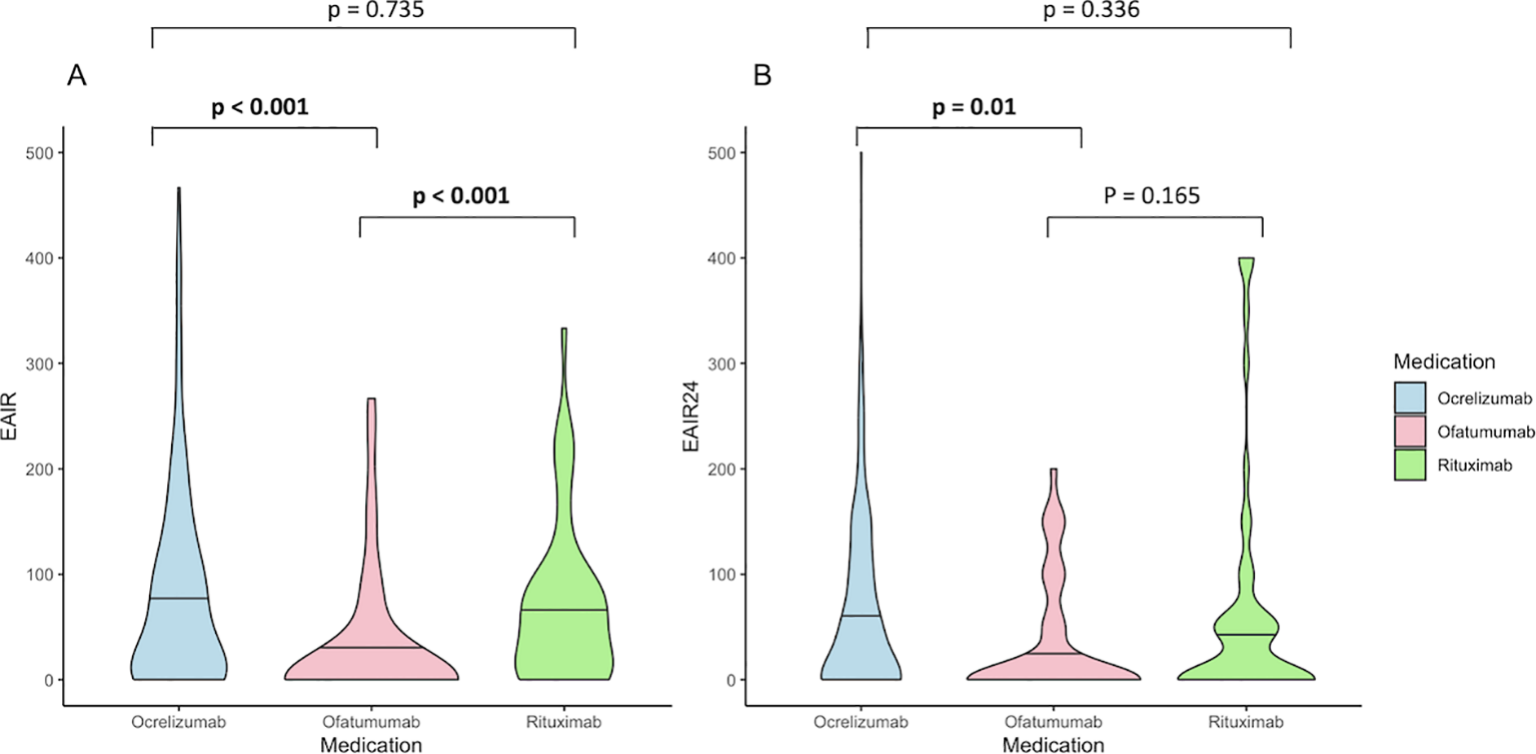
Violin plots showing infections under B cell depletion (BCDT): exposure-adjusted incidence rate per 100 patient-years (EAIR) calculated based on **(A)** the whole follow-up time and **(B)** for the 24 months of treatment (EAIR24), respectively. Horizontal lines display the mean EAIR/EAIR24. P-values were calculated by use of the Kruskal-Wallis test, and the Bonferroni test was used for *post hoc* analysis.

EAIR was calculated separately for all three substances, followed by the performance of Kruskal-Wallis tests to detect drug-specific differences. The mean EAIR over the entire observation period was significantly lower for OFA (29.0) than for RTX (68.7) and OCR (81.0, both p < 0.001, **Figure 3A**). To avoid distortions due to the longer treatment duration for RTX and OCR compared to OFA, the EAIR was also calculated from the number of infections over the first 24 months of treatment (EAIR24). This still resulted in a significantly lower mean EAIR24 for OFA (31.8) compared to OCR (63.0, p = 0.010); the difference was no longer detectable compared to RTX (p = 0.165, **Figure 3B**).

When looking at bivariate correlations, longer duration of treatment was found to be significantly associated with a higher EAIR (p = 0.003), while age at the beginning of treatment (p = 0.916) and baseline EDSS (p = 0.690) had no association. Patients with PPMS who received OCR treatment exhibited a trend towards higher EAIR compared to those with RMS (Mann-Whitney U’s p = 0.064).

We also performed a multiple regression analysis to verify the influence of the respective drugs on EAIR. After taking into account the potential confounders, follow-up time, baseline EDSS, age at the start of therapy, and the presence of IgG hypogammaglobulinemia during therapy, medication choice remained a statistically significant factor influencing infection frequency (p = 0.012).

### Hypogammaglobulinemia

#### IgG

**Figure 4:**
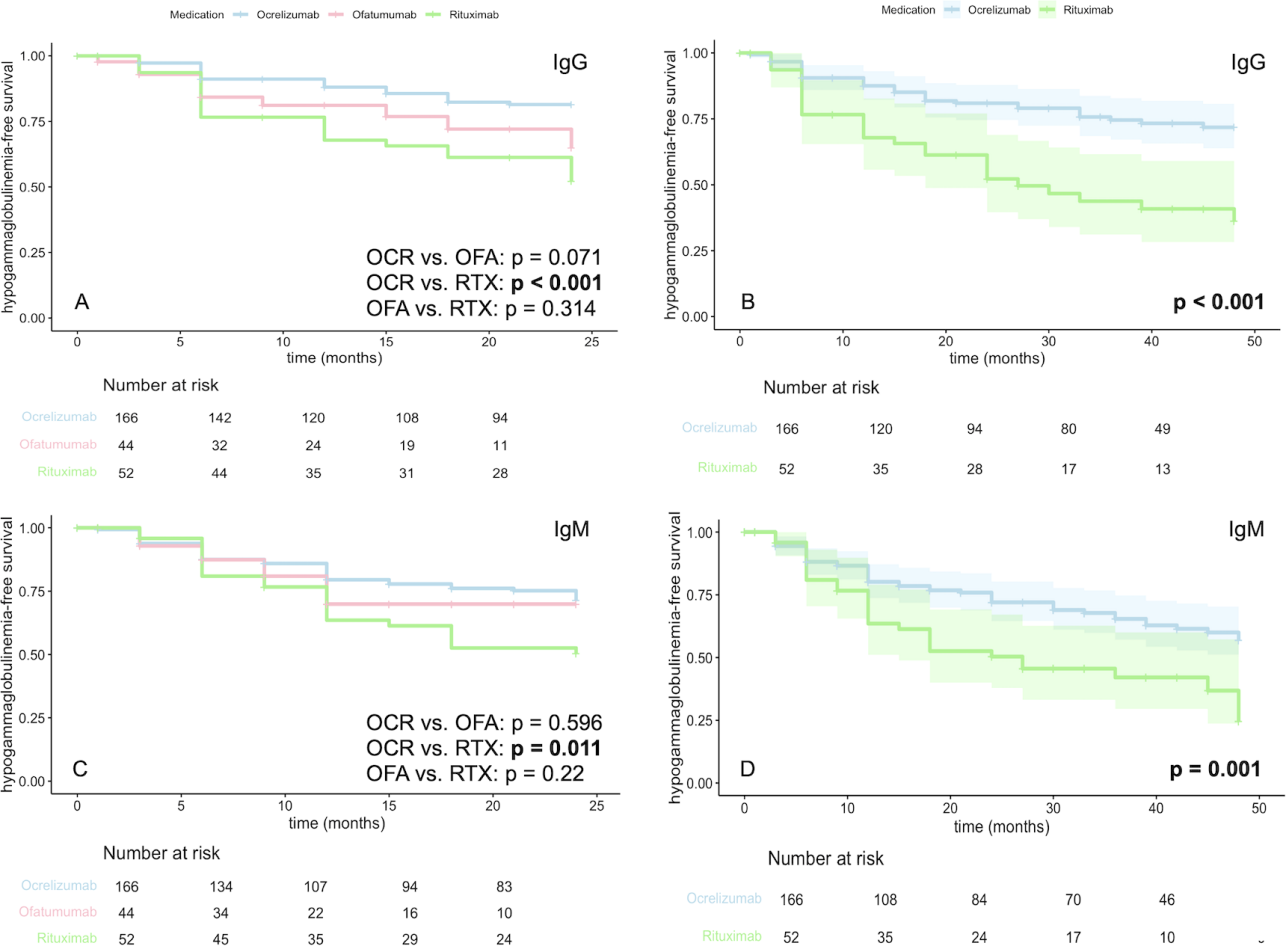
Kaplan–Meier plots indicating the proportion of patients without evidence of hypogammaglobulinemia. Shaded areas display 95% confidence intervals. Comparison of IgG hypogammaglobulinemia **(A, B)** in patients with OCR, OFA, and RTX over 24 months **(A)** and in patients with OCR and RTX over 48 months **(B)**. Comparison of IgM hypogammaglobulinemia **(C, D)** in patients with OCR, OFA, and RTX over 24 months **(C)** and in patients with OCR and RTX over 48 months **(D)**. OCR, Ocrelizumab; OFA, Ofatumumab; RTX, Rituximab.

Results of the hypogammaglobulinemia analysis are summarized in **Supplementary Table 1**; hypogammaglobulinemia-free survival is visualized as Kaplan-Meier curves in **Figure 4**.

IgG hypogammaglobulinemia (IgG < 700 mg/dL) was identified in 57 out of 262 patients (21.8%) following a maximum of 24 months of monitoring. Patients with an IgG deficiency prior to therapy start, which did not worsen during therapy, were not included in the hypogammaglobulinemia group. We found that 15.1% of patients under OCR (n = 25), 22.7% under OFA (n = 10), and 42.3% under RTX therapy (n = 22, p < 0.001) developed hypogammaglobulinemia. The RTX cohort demonstrated a significantly faster development of hypogammaglobulinemia in comparison to the OCR cohort (p < 0.001, **Figure 4A**).

For OCR and RTX, large data collections were available for a follow-up of 48 months, with 20.5% of OCR patients (n = 34) and 51.9% of RTX patients (n = 27) being identified as developing IgG deficiency during this period (p < 0.001). In this cohort, RTX patients also exhibited a significantly faster onset of hypogammaglobulinemia (p < 0.001, **Figure 4B**).

The presence of IgG hypogammaglobulinemia was not associated with older age at therapy initiation (p = 0.886). Patients with IgG-hypogammaglobulinemia exhibited a higher mean EAIR24 (66.7 vs. 55.4), though this did not reach statistical significance (p = 0.263).

To confirm our findings, we added a multiple binary logistic regression model. After correcting for age, follow-up time, and baseline IgG values, the association between medication choice and hypogammaglobulinemia remained statistically significant, with rituximab (RTX) being more frequently associated with hypogammaglobulinemia than the other substances (p < 0.001).

#### IgM

When examining a maximum of 24 months since the commencement of the BCDT, IgM hypogammaglobulinemia (< 40 mg/dL) was found in 70 of the 262 patients (26.7%). With regard to the individual drugs, we observed an IgM deficiency in 22.3% of OCR patients, 22.7% of OFA patients, and 44.2% of RTX patients (p = 0.006). Within a maximum of 48 months of follow-up, hypogammaglobulinemia was identified in 28.9% of patients treated with OCR and in 55.8% of those treated with RTX (p < 0.001). The RTX cohort demonstrated a significantly faster development of IgM hypogammaglobulinemia compared to the OCR cohort, both during the initial 24-month therapy period (p = 0.011, **Figure 4C**) and across the entire 48-month maintenance therapy period (p = 0.001, **Figure 4D**).

In bivariate correlations, the presence of IgM hypogammaglobulinemia was not significantly associated with the age of the patients (p = 0.891), nor with a higher mean EAIR24 (p = 0.701) or EAIR (p = 0.154).

### Immune cell dynamics

Flow-cytometric phenotyping was performed in all patients included in this study. Laboratory follow-up for the OFA cohort was available quarterly for a period of 24 months and for the OCR and RTX cohort for a period of 48 months. CD19^+^ B cells remained at a median of 0% under treatment with all anti-CD20 antibodies.

### Ocrelizumab

Based on total white blood cell counts, patients undergoing OCR therapy demonstrated a significant decrease in the median percentage of lymphocytes (p = 0.016, R^2^ = 0.33), along with a trend towards a decrease in the median percentage of eosinophils (p = 0.070). In contrast, there was a slight increase in the proportion of neutrophils. Likewise, there were no significant changes in basophils and monocytes. The proportion of CD3^+^ T cells increased significantly (p = 0.013, R^2^ = 0.34), whereas the median percentage of CD4^+^ and CD8^+^ cells, the CD4/CD8 ratio, or the median proportion of NK cells did not change (p > 0.05 in each case).

### Ofatumumab

Among leukocyte populations, basophils showed a trend towards an increase (p = 0.081) with no other therapy-associated changes. We detected a significant increase in the median proportion of CD4^+^ cells (p = 0.021, R^2^ = 0.55) and the median CD4/CD8 ratio (p = 0.002, R^2^ = 0.76). In contrast, the median proportion of CD8^+^ cells tended to decrease, although this decrease was not statistically significant. Likewise, no dynamic changes in the percentage of CD3^+^ T cells and NK cells were observed.

### Rituximab

In RTX patients, there was a trend towards an increase in basophils (p = 0.082); beyond this, there were no relevant therapy-associated immune cell dynamics.

**Figure 5:**
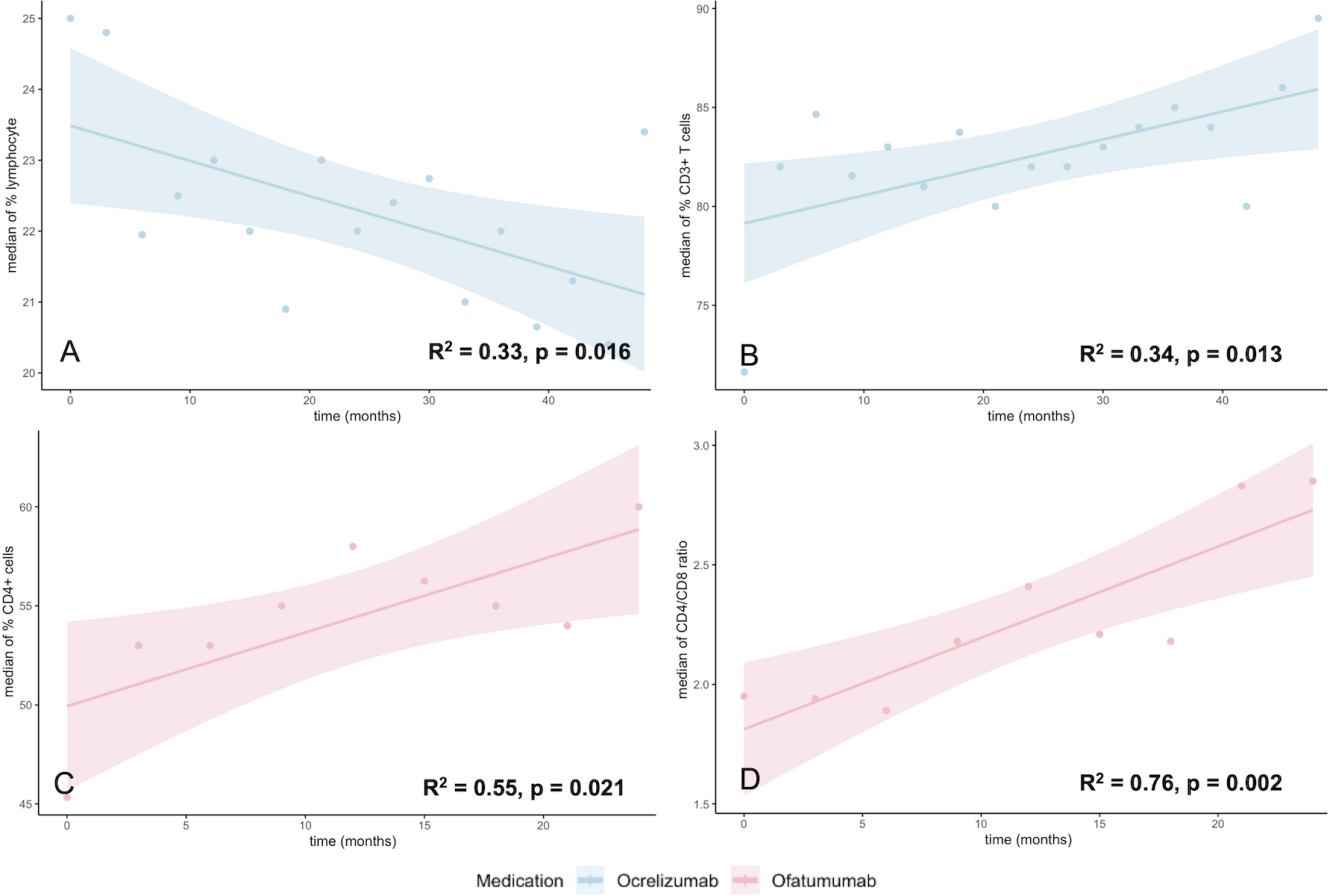
Significant immune cell dynamics of patients treated with OCR (blue) and OFA (light red). Each dot represents median cell counts of the whole cohort (OCR n = 166, OFA n = 44): Course of the lymphocyte percentage **(A)** and CD3 T cell percentage **(B)**, both in OCR. Course of CD4 T cell percentage **(C)** and CD4/CD8 ratio **(D)**, both in OFA.

When looking at the moderator analysis, a significant effect of the medication on the cell counts was only found for the above-mentioned correlations (OCR: Lymphocytes (p = 0.040), CD3 T cells (p = 0.038); OFA: CD4 (p = 0.024, CD4/CD8 (p = 0.008)). This finding underscores the relevance of the observed effects. The most important results of the single substances are illustrated in **Figure 5**. Direct head-to-head comparison of the significant results is displayed in **Supplementary Figure 1**.

### Absolute cell numbers

In addition to the dynamics of immune cell frequencies, we also performed calculations using absolute cell numbers. This confirmed the increase in CD3^+^ T cells under OCR (p = 0.036, R^2^ = 0.262), mediated by an increase in CD4^+^ cells (p = 0.004, R^2^ = 0.427). The decrease in lymphocytes under OCR (p = 0.371) and the increase in CD4^+^ T cells under OFA (p = 0.288) were also observed, but without reaching statistical significance. Please note that the dynamics of the CD4/CD8 ratio were not recalculated, as this is a ratio value. No additional significant correlations were identified for the other cell populations.

### T cell function

**Figure 6:**
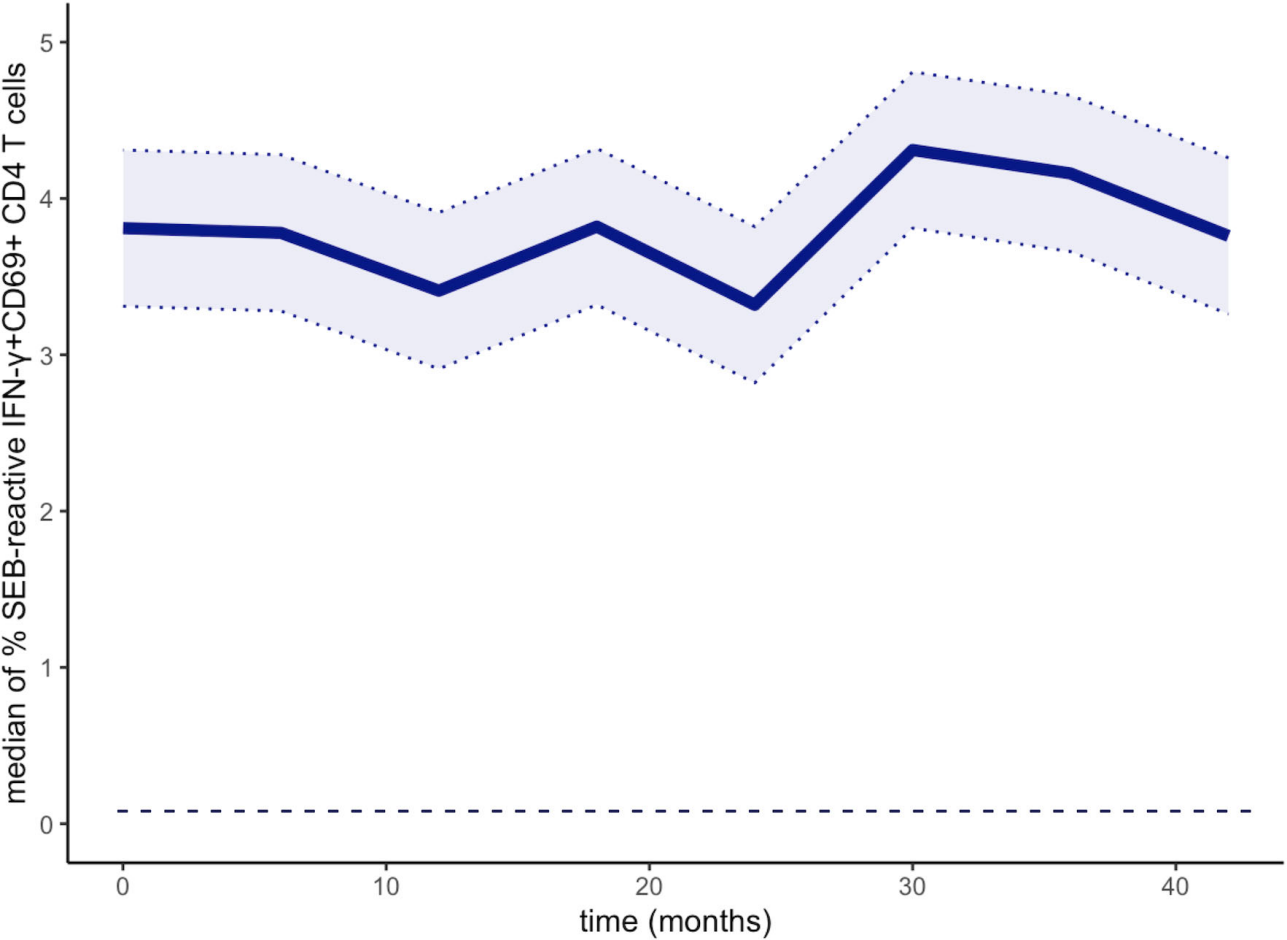
Median percentage of SEB-reactive (IFN-γ CD69^+^) CD4 T cells during the follow-up period for n = 151 patients (OCR n = 91, OFA n = 37, and RTX n = 23). The detection limit of 0.05% is displayed as a dashed line. SEB, *Staphylococcus aureus* enterotoxin B.

The percentage of functional CD4^+^ T cells after stimulation with *SEB* was quantified at 6-monthly intervals. Results from a subgroup of 151 patients were available for this analysis (OCR n = 91, OFA n = 37, and RTX n = 23). Therefore, a direct comparison between the substances could not be made. As shown in **Figure 6**, the median level of SEB-reactive CD4 T cells, characterized by coexpression of IFN-γ and CD69, remained stable for 48 months of BCDT, indicating that T-cell reactivity was preserved under long-term anti-CD20 therapy. Following stimulation with the negative control (PBS), no reactive T cells were measured (threshold 0.05%).

## Discussion

In the present study, we performed a longitudinal clinical and immunological characterization of three cohorts undergoing BCDT using OCR, OFA, and RTX. A direct comparison of the different substances regarding the various clinical and paraclinical parameters was obtained.

When comparing the three substances, it is important to bear in mind that RTX and OCR are infusion therapies, and one knows that patients receive the full dose. In contrast, OFA is self-administered, and knowledge of patient dosage is not necessarily possible. This may provide a partial explanation for the observed differences in tolerability and biological effects. However, median percentage of CD19^+^ B cells among all three agents was 0% from the first visit after initiation, indicating a complete therapeutic effect.

Research and clinical experience demonstrate the effectiveness of both OCR and OFA in preventing relapses in RMS (7, 14, 15, 19, 31). This observation pertains to RTX, despite its off-label utilization (10, 11). OCR and RTX are furthermore useful in PPMS patients (12, 35). ARR of OCR/OFA was comparable to that described in other studies (19, 36–38) without significant differences between the substances. This finding is consistent with previous research (39). During the observation period of more than 100 weeks, the large majority of patients remained relapse-free. Median EDSS increased with both substances. This is most likely due to progression independent of relapse activity (PIRA), given that the RMS patient cohorts also included patients with aSPMS. The findings underscore the high potency of both drugs.

Infections are the most common adverse events associated with BCDT (18, 19). We defined clinically relevant infections as a combination of clinical symptoms and an increase in laboratory infection parameters. Infections detected like this therefore had implications for the patient’s daily life. Previous studies defined infections according to patients’ reports, potentially resulting in an higher infection rate (16, 17). In our cohort, we observed a significantly lower EAIR with OFA compared to the other two substances. Infections were the most common reason for discontinuation of therapy in OCR patients. Cohort-specific characteristics, such as the presence of PPMS or age at therapy initiation were not a significant influencing factor. Therefore, a substance-specific effect must be assumed. The duration of immunosuppression, on the other hand, was a relevant factor, and the follow-up times of RTX and OCR were significantly longer than those of the OFA cohort. Consequently, we calculated an infection rate from the first 24 months of therapy. In these analyses, EAIR of OFA (31.8) was also significantly lower than that of OCR (63.0). It should be noted that other studies did not make such an adjustment (15, 17, 19). Overall, OFA appears to have a relevant advantage with regard to the risk of clinically relevant infections.

To understand the underlying mechanisms, an analysis of the dynamics of immune cells and immunoglobulins was performed. All BCDT agents can result in the development of secondary hypogammaglobulinemia (18, 20, 23, 24, 40). In the past, it was postulated that IgG levels remain stable under OFA (41). However, there is evidence that these findings were partly due to the design of the authorization studies (7, 19). We observed IgG and IgM deficiency in our cohort for each substance, with no differences between OFA and OCR. RTX patients, on the other hand, developed hypogammaglobulinemia more frequently and rapidly, with more than 50% of patients affected at least once after 48 months. The extent of the respective Ig deficiencies may be explained by the different penetration of the monoclonal antibodies into B-cell-containing compartments, as well as the different mechanisms of mediated cytotoxicity (42). However, we did not observe a significant correlation between hypogammaglobulinemia and infection rate. The literature contains a variety of data regarding this relationship, some of which appear to be in line with our results (23, 43–45), while others appear to contradict them (46, 47).

Overall, hypogammaglobulinemia is unlikely to be the only factor leading to infection during BCDT. We therefore analyzed the dynamics of immune cell subpopulations. In order to avoid distortions regarding fluctuations in total leukocyte count, we analyzed percentages, which makes our results more robust. However, it should be noted that the majority of these findings were also applicable to absolute cell counts. OCR patients demonstrated a substantial decline in the proportion of lymphocytes. This may explain why these had the highest infection rates despite lower rates of hypogammaglobulinemia. As per the literature, lymphopenia develops in approximately 20% of cases under OCR (25, 48, 49). While the available data set is limited, initial findings suggest that the incidence of this is lower with OFA (15, 50, 51). In line with this, we did not see a significant decrease in the median lymphocyte count under OFA. This finding could be responsible for the higher infection rate in the OCR cohort. In OFA patients, we observed a significant increase in the CD4/CD8 ratio. This effect was not observed to the same extent in the OCR and RTX groups. Previous studies have not identified any significant differences between the substances in terms of the effect on CD4^+^ or CD8^+^ T cells, although the sample sizes have been smaller (52). Data on the effect of OFA therapy on the distribution of CD4^+^ and CD8^+^ T cells and its clinical implications require further investigation. One possible explanation for the increase in the CD4/CD8 ratio is the depletion of CD20+ T cells, which are enriched in CD8+ cells (53, 54). Further investigation of this correlation is required.

CD20^+^ B cells are key players in both the humoral and the cellular immune response, through their interaction with T cells. To date, there is a paucity of data on the functionality of T cells under BCDT. A study of rheumatological patients provided evidence of impaired T cell function (29). In the past, some authors have discussed that influencing the interdependence between B and T cells accounts for a significant part of the effectiveness of medications that deplete B cells (55). We demonstrated stable levels of SEB-reactive IFN^+^CD69^+^ CD4 T cells over the entire observation period. This indicates sufficient T cell function despite ongoing BCDT.

## Limitations

The main limitation of this study is the heterogeneity of the cohorts. Firstly, the follow-up period for patients treated with OFA was shorter. Secondly, RTX patients had a variety of diagnoses and a higher age than those in the other groups. However, age was not a significant influencing factor for the development of infections or hypogammaglobulinemia, so the observed differences can be categorized as relevant. The same was true for the analysis of infection rates. Multivariable models confirmed the RTX-induced effect in both subjects. Nevertheless, it is clear that further comparisons including RTX-treated MS patients are necessary, as the results obtained can only be used to generate hypotheses for future studies. PPMS patients under OCR did not demonstrate a higher infection rate than RMS patients, which is why this heterogeneity is also negligible. Finally, the OCR patients had higher baseline EDSS values than the OFA patients, but this was not a significant confounding factor with regard to the risk of infection, either.

When considering hypogammaglobulinemia, it should be noted that follow-up over 24 or 48 months was not available for all patients. Nevertheless, significant differences between the different substances were identified.

## Conclusions

In this study, we characterized the clinical and immunological status of patients undergoing long-term BCDT on a longitudinal basis. A series of systematic head-to-head comparisons was conducted between the substances.

The mentioned findings may help to guide future individual treatment decisions and patient counselling.

## Data Availability

The raw data supporting the conclusions of this article will be made available by the authors, without undue reservation.

## Glossary

AE: Autoimmune encephalitis
ARR: Annual relapse rate
aSPMS: Active secondary progressive multiple sclerosis
BCDT: B cell depletion treatment
CD: Cluster of differentiation
CI: Confidence interval
CRP: C reactive protein
EAIR: Exposure-adjusted incidence rate
EDSS: Expanded disability status scale
e.g.: Example given
IFN: Interferon gamma
IgA: Immunoglobulin A
IgG: Immunoglobulin G
IgM: Immunoglobulin M
OCR: Ocrelizumab
OFA: Ofatumumab
MS: Multiple sclerosis
NK: Natural killer cells
PBS: Puffered Based Saline
PIRA: Progression independent of relapse activity
PPMS: Primary progressive multiple sclerosis
RMS: Relapsing multiple sclerosis
RRMS: Relapsing remitting multiple sclerosis
RTX: Rituximab
SEB: Staphylococcus aureus enterotoxin B.
