# Supplementary material for: Real-world comparison of anti-CD20 therapies: efficacy, infections, and immune profiles in a German cohort
Source: Front Immunol. 2026 May 12;17:1738865. doi: 10.3389/fimmu.2026.1738865 (PMC13201113; doi:10.3389/fimmu.2026.1738865)
Supplement: Supplementary file 1 [file SupplementaryFile1.docx]

**Supplement**

|  | OCR (n = 166) | OFA (n = 44) | RTX (n = 52) | all | p* |
| --- | --- | --- | --- | --- | --- |
| 24 months follow-up  IgG Hypogammaglobulinemia  yes / no, *n (%)*  IgM Hypogammaglobulinemia  yes / no, *n (%)* | 25 / 141  (15.1 / 84.9)  129 / 37  (77.7 / 22.3) | 10 / 34  (22.7 / 77.3)  34 / 10  (77.3 / 22.7) | 22 / 30  (42.3 / 57.7)  29 / 23  (55.8 / 44.2) | 57 / 205  (21.8 / 78.2)  70 / 192  (26.7 / 73.3) | **< 0.001**  **< 0.001** |
| 48 months follow-up  IgG Hypogammaglobulinemia  yes / no, *n (%)*  IgM Hypogammaglobulinemia yes / no, *n (%)* | 34 / 132  (20.5 / 79.5)  48 / 118  (28.9 / 71.1) | NA  NA | 27 / 25  (51.9 / 48.1)  29 / 23  (55.8 / 44.2) | 61 / 157  (28 / 72)  77 / 141  (35.3 / 64.7) | **< 0.001**  **< 0.001** |

**Supplementary Table 1:** Hypogammaglobulinemia under B cell depletion. IgG = immunoglobulin G, IgM = immunoglobulin M, OCR = Ocrelizumab, OFA = Ofatumumab, RTX = Rituximab, NA = not available.

*p values were calculated via chi square test.

**Supplementary Figure 1:** immune cell dynamics of patients treated with OCR (blue), OFA (light red) and RTX (green. Each dot represents median cell counts of the whole cohort (OCR n = 166, OFA n = 44, RTX = 52): Course of the lymphocyte percentage (A), CD3 T cell percentage (B), CD4 T cell percentage (C) and CD4/CD8 ratio (D).


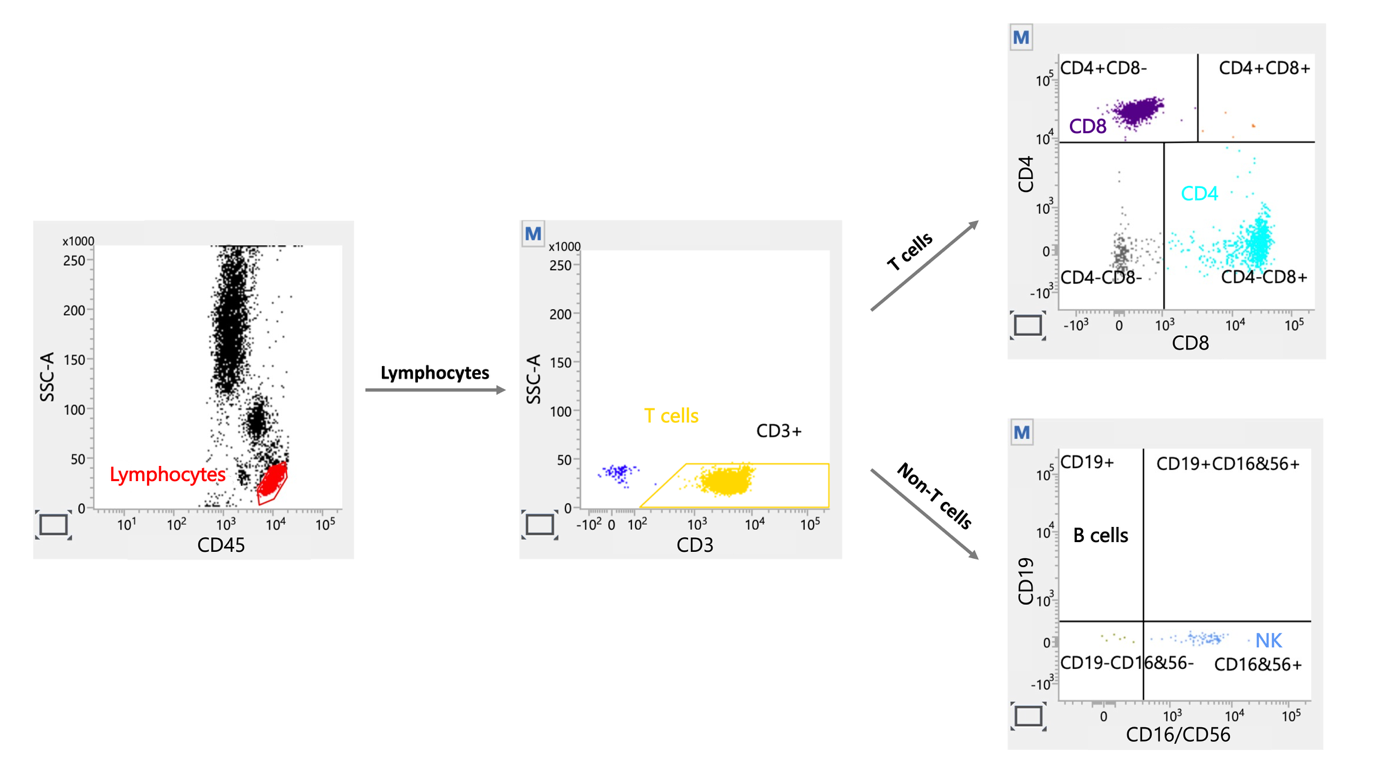


**Supplementary Figure 2:** Flow cytometric analysis of immune cell phenotyping. First, all measured events were shown in SSC (side scatter) and were displayed for CD45 to determine lymphocytes. These were subsequently displayed for CD3 to determine T cells. CD3^+^ T cells were finally displayed for CD4 and CD8 to determine T cell subpopulations, while CD3^-^ cells were displayed for CD19 and CD16/CD56 to determine B cells and natural killer cells (NK). Please note that the representative image was taken from a patient under B cell depletion treatment, leading to a non-existence of CD19^+^ B cells.


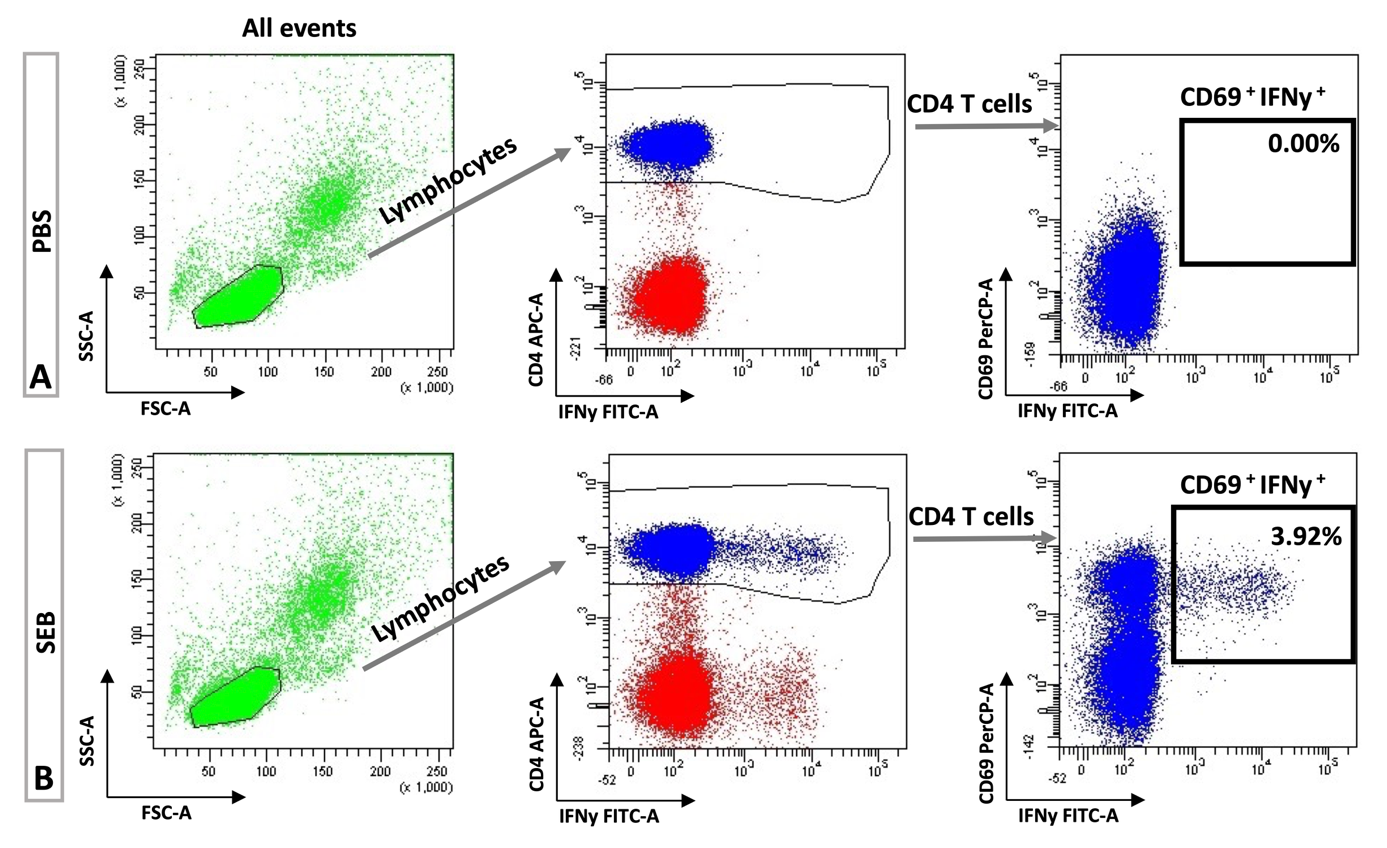


**Supplementary Figure 3:** Antigen-reactive CD4 T cells were identified by intracellular staining and flow cytometry analysis after whole blood stimulation with PBS (Phosphate Buffered Saline, **A**) and SEB (Staphylococcus aureus Enterotoxin B, **B**). First, all measured events were shown in FCS (forward scatter) and SSC (side scatter) to determine lymphocytes, which were further displayed for CD4 and IFNγ expression. CD4+ T cells were subsequently displayed for CD69 and IFNγ expression to quantify the percentage of CD69+IFNγ+ cells.
